# Women Who Love: An Explorative Study on Experiences of Lesbian and Bisexual Women with a Mild Intellectual Disability in The Netherlands

**DOI:** 10.1007/s11195-018-9519-y

**Published:** 2018-02-01

**Authors:** J. M. T. Stoffelen, D. Schaafsma, G. Kok, L. M. G. Curfs

**Affiliations:** 1 Governor Kremers Centre, Maastricht University, Maastricht, The Netherlands; 2 Psychology and Neuroscience, Work and Social Psychology, Applied Social Psychology, Maastricht University, Maastricht, The Netherlands; 3 Zorgbelang Gelderland-Utrecht, Arnhem, The Netherlands; 4 Fontys University of Applied Sciences, Eindhoven, The Netherlands

**Keywords:** Homosexual, Gay, Lesbian, Bisexual, Women, Sex, Intellectual disability, Learning disability, Sexual behaviour, Sexual rights, Support needs, Netherlands

## Abstract

Empirical research that addresses sexual orientation in people with an intellectual disability (ID) is limited, and very little is known regarding the personal experiences of lesbian and bisexual women with ID. This study set out to answer the question: *What are the experiences of lesbian and bisexual women with a mild intellectual disability in the Netherlands?* Ten lesbian and bisexual women (average age of 33 years) with a mild intellectual disability took part in our study comprising of semi-structured interviews. Participants reported that they had found it hard to talk to others about sensitive subjects such as their sexuality, and had been left to figure out information regarding their sexual orientation without support or guidance. Our results point to a lack of information, sexual education and role models when it comes to lesbian sex and women with an intellectual disability. Social contact was often limited, and participants experienced difficulties finding a partner. Furthermore, participants often had to cope with mental health problems and had struggled with loneliness, depression and addiction. Last but not least, our participants reported that they had been discriminated against. Coming out (revealing your sexual orientation) is not easy when you have an intellectual disability. To enable women with ID who have lesbian or bisexual feelings to understand and secure their sexual rights in their daily lives is important. Therefore, it is necessary to provide support in the following domains: sexual education and training, social contact and assertiveness.

## Introduction

### Sexual Rights

People with an intellectual disability (ID) have the same human rights as everyone else [1], and therefore also the same sexual rights. A working definition of sexual rights has been formulated and established by the World Health Organization [2]. These rights—for example to choose a partner, to equality, to liberty, to non-discrimination, to privacy, to the freedom of thoughts, opinions and expression—are guiding principles. In 2006, the United Nations adopted The Convention on the Rights of Persons with Disabilities [3], which recognized the equal and inalienable rights of people with a disability. Article 25 includes ‘the area of sexual and reproductive health’ and ‘require(s) health professionals to provide care of the same quality to persons with disabilities as to others, including on the basis of free and informed consent by, inter alia, raising awareness of the human rights, dignity, autonomy and needs of persons…’. The American Association on Intellectual and Developmental Disabilities (AAIDD) [4] has also acknowledged the sexual rights of people with an ID: ‘These rights and needs must be affirmed, defended, and respected’. Sexual orientation is one of the rights mentioned in the Position Statement of the AAIDD. Unfortunately, these rights are not always self-evident for people with ID [5, 6]. Often, individuals with an ID do not get the respect, or the necessary support, to achieve their sexual rights.

### Women with ID

Studies on sexuality in general among women with ID are also limited, and the results often describe negative perceptions, negative experiences, and failures in sex education: ‘disempowerment is woven into every strand of the lives of many women with intellectual disability’ [7]. Research from the perspective of women with ID who have sexual feelings for other women is very underrepresented in scientific studies [8–15].

### LGBT with ID

Lesbian women, gay men, bisexual individuals and transgender individuals (LGBT) without an ID experience an increased risk for health problems such as depression, anxiety disorders, drug and alcohol abuse, loneliness, sexual problems, and an increased risk of sexual violence [16]. Research into the specific health problems among LGBT people with intellectual disabilities is scarce [11, 15, 17]. In a review of the literature, McCann et al. [15] included 14 papers that addressed the views, issues, concerns of people with ID who identified as LGBT. More awareness of the interests of LGBT individuals with ID is needed. Reducing stigma and discrimination can improve the health and well-being of LGBT individuals [15]. In the existing literature, we found that three important themes emerged in relation to LGBT individuals with ID: stigma, social contact and support.

#### Stigma

Gay, lesbian, and bisexual individuals with an ID experience prejudice and harassment from the outside world. They often have to deal with the double stigma associated with their intellectual disability and their sexual orientation. This has also been referred to as ‘layered’ stigma [11, 18, 19]. Experiencing prejudice and discrimination can limit an individual’s opportunities to participate in society, and limit opportunities to develop friendships [20]. LGBT individuals with intellectual disabilities often lack social and financial power [21]. Moreover, they are more likely to experience exclusion, discrimination and harassment [10–12, 14, 22–24]. Peers with ID often have prejudices and negative attitudes towards homosexuality. Young people with ID sometimes internalise the homophobic and heterosexual attitudes of nurses, teachers and parents [11]. Gay men, lesbian and bisexual individuals with ID find it difficult to be open about their sexual orientation [8, 9, 11, 14, 25].

#### Social Contact

Many people meet their partners at work, whilst pursuing leisure-activities, or via their social network. Gay men, lesbian and bisexual individuals with ID experience difficulties when it comes to finding sexual partners. Their social network is often limited, and they are often stigmatized; this reduces the chance of finding a partner [8, 14, 17]. A literature search carried out by McCarthy [7] confirms this, and shows that there are yet more obstacles for women with ID, including low self-esteem, a lack of assertiveness needed to set sexual boundaries, and a lack of sex education to emphasise the enjoyable side of sex and to provide information about dealing with risky situations. In the Netherlands, LGBT individuals with ID find it hard to meet up with friends due to their limited mobility [14]. Lack of mobility is a big problem for people with a disability [26], and one that can prevent them from leading an independent life. To meet others is important. For example, in order to experience relationships, to develop a positive self-image, and become more assertive. The internet can offer an alternative to meeting people in person [17], although the experiences of people with ID using the internet are not always positive [14].

#### Support

Some issues deserve special attention when it comes to supporting LGBT individuals with ID, namely sex education and training, and the provision of positive support, role models, and accessible information. Sex education and training is not only necessary for individuals with ID but also for the individual’s support network and family. Sexual diversity must be included in the programme [11, 14, 22, 27, 28], along with a number of items and issues such as prevention of HIV/AIDS, homophobia, sexual orientation, and having access to the LGBT community [11, 14, 17, 28, 29]. The provision of positive support and role models is important in terms of the development of a positive identity [8, 11, 12, 14, 22, 28]. It is essential to provide accessible information about LGBT sexuality, for example via good “easy to read” books, websites, and photo-stories [11, 12].

### Aim and Research Questions

To date, the sexual experiences of women with ID have received scant attention in research, and little is known of the personal experiences of lesbian and bisexual women with ID. The additional (sexual) vulnerability of women with ID in general underlines the need for a study among this specific group. In this exploratory study, our aim is to gain insight into the lives of a specific cohort of women with ID who have sexual feelings for other women, or who identify themselves as lesbian or bisexual, and are living in the Netherlands. Through in-depth interviews, the following topics were addressed: *What are the experiences of lesbian and bisexual women with a mild intellectual disability in the Netherlands?* Furthermore, we asked the following sub-questions: *What positive or negative experiences do these women encounter? What are the (support) needs of these individuals, and what problems do they experience?*

## Methods

### Participants

Participants were lesbian or bisexual women with mild ID. Thirteen women were invited to participate in the study, and of these, three decided not to participate. The participants were recruited through intermediaries of associations that support people who are diagnosed with an ID and sexologists who work with people with ID. These intermediaries, sexologists and the researchers explained the research to potential participants. All participants received a leaflet containing information about the research: our research goals, the questions addressed in our research, as well as information about confidentiality and support. Participants were asked to contact the researchers themselves if they wanted to participate in the research. All participants were adults. The mean age was 33 years with an age range of 25–47 years (Table 1). The women lived in various provinces all over the Netherlands. The process of finding participants was intensive. Women with an ID who are lesbian or bisexual seem to be invisible, in that they are often not recognised or noticed, even by their own support workers.

**Table 1:** Summary of selected demographic information of participants

Demographic variable	Description	N = 10
Age	20–29	4
	30–39	4
	40–49	2
Ethnicity	Native Dutch	10
Residential placement	Living independently with support	3
	Own apartment in a care complex	6
	Living in a care complex	1
Marital status	Single	6
	In a relationship	2
	Married	2

### Informed Consent

Approval of the Ethical Review Committee Psychology and Neurosciences at Maastricht University was obtained before the start of the study. Several guidelines for conducting research with people with an ID have been developed [e.g., 30–32]. In this study, we used the ‘informed consent procedure’ for participants with an ID developed by Thomas and Kroese [32]. In following this procedure, participants are not only informed about the research but they also have to answer a few questions in order to indicate that they have understood the information received. Information about confidentiality, reporting sexual abuse, anonymity, and working with recording equipment was included [27]. Attention was drawn to the participant’s right to withdraw at any stage of the data collection. All participants were able to understand this information, decided to participate in the study, and signed the consent forms. Participants were told that if they felt that they wanted additional support after the interview, they could meet with an independent qualified counsellor (a psychiatrist with specific expertise in people with ID).

### The Interview

Participants were asked about their sexuality in an explorative semi-structured interview, using a topic list of interview questions (see “Appendix” section). The list was developed using input from professionals in the field of ID and in conjunction with members of the target population. Two pilot interviews were conducted. These interviews are included in this study. The interviews lasted approximately one hour, depending on participants’ ability to concentrate.

### Analysis

The interviews were audio-taped. Recorded interviews were typed out verbatim, and the transcripts were analyzed using NVivo version 10. For analysis, a three-step approach was applied. In step 1, paragraphs of individual transcriptions were given codes, which were basically short descriptions of the contents of those paragraphs. In step two, categories were identified from the list of codes. Codes that were similar were merged. Step 3 consisted of identifying subcategories within the major categories. Analyses were conducted by the first author.

## Results

In total, eight interviews were conducted with 10 women with an ID. Two participants were interviewed together with their partner (2 × 2 participants); six participants preferred an individual interview. The following topics were identified and are presented in the following sections: support, coming out, sexual experiences, mental health, social contact, and discrimination.

### Support

Eight participants talked about the support they had received. This support came from several sources and was related to different life domains: finance and administration, mental wellbeing, physical health, and household matters. The participants had to interact with all sorts of support workers, including supervisors, trainees, personal coaches, and work assistants. Often, these people provided support once a week for a short period of time. As a result, some participants found it difficult to build a relationship of trust with these support workers, and to talk to them about sensitive subjects like sex. They must often seek information without any support or input from others.

No, I don’t talk to them about it (sexuality). It’s none of their business… They don’t stop talking about it… There are almost no men, only women. When I go to my doctor I don’t talk about it either. And he doesn’t ask me about it. (34 years)

Well, I have my own coach. I never discussed it (sexuality). We never talk about sex…. They are curious. That’s what they are. They don’t have time. They have half an hour…One is always in a hurry. Then I just think: go away… I want my one support worker, not an intern. (47 years)

However, six participants reported that they were content with the professional support workers, who helped them find their way in the LGBT-community, and made them feel safe and accepted.

My mentor, he is the best. I picked him myself. He is also gay. That makes you feel safe…. (40 years)

Who is a good support worker? Someone who helps me find my way. So I do not have to figure out everything alone. (26 years)

### Coming Out

Six out of ten participants talked about their coming out experiences; the age at which they came out ranged from 13 years to adulthood. Some women had been supported in the process of coming out; others had been left to deal with it alone. All women felt insecure about themselves, had to deal with being verbally unskilled, and were not used to standing up for themselves.

I knew it from my 15^th^ and kept it secret. My uncle was killed because he was gay. Also nowadays, I keep it secret, sometimes. It’s just hard, sometimes…. Many women are anxious when it comes to telling the outside world who you are. I did it because I want to support people who are still afraid. I want to be a role model for them. (40 years)

From childhood… How shall I say it…? I knew it but it was not allowed. I met my husband and had children… Then I saw her and I thought: now I am sure! All those years I hadn’t thought about it… (47 years)

A homosexual friend said: You have to learn to speak up for yourself. I know you are homosexual. You are ready for it! (38 years)

I knew it since I was a child…during my teenage years. I had butterflies in my stomach when I saw women. (36 years)

I found it very difficult to tell my father that I am a lesbian. I thought he would become angry. (25 years)

Nine participants talked about how their friends had responded to their coming out. One woman reported that this news had elicited negative reactions, one woman had experienced a combination of negative and positive reactions, and seven women had experienced only positive reactions. For all of them, it was nerve-racking to tell friends about their sexual orientation. Most women didn’t know other lesbian or bisexual women.

We know few other women like us. (47 years)

Among my friends, I’m the only one who is gay. (25 years)

In contrast with the other participants, one couple appeared not to be an exception in their network of friends and family:

All our friends…almost everyone is gay… oh, no, one is bisexual. (38 years)

Finding a partner was an issue for six women. Two participants indicated that they hoped to find a friend online, and two women thought that they might find a partner at meeting places offered by the COC.^1^ Two women reported that they did not know how to find a partner.

Where can I find them? (26 years)

If you search in a forced way, you get weird people. You have to wait till you find someone you really like. (25 years)

### Sexual Experiences

All participants spoke openly about their sexual experiences: eight women had sexual experience—all with both men and women—and two women had not had any sexual encounters. These eight women reported that they’d had to find out almost everything by themselves when it came to having sex with another woman.

The first time, everything was very strange. It’s very different with women. We don’t have videos that show how to do it. I never had solo sex. My girlfriend knew how to do this. I found it very strange. I had to learn it. (25 years)

I don’t have experience with sex. I have kissed with a boy. And I didn’t like it. In terms of sex, I have no experience. (25 years)

Sex with a woman is different. It is no longer an obligation every evening…. now it’s fine and a pleasure. It is more enjoyable (than with men). (47 years)

I didn’t know how to reach sexual climax. I was curious…. And it was fine. (40 years)

Someone who was in love with me, stalked me. That made me very anxious. I didn’t dare to pick up the telephone anymore. It was hard to talk about it with others. Fortunately, it happened a long time ago. I have never had sex with women. Nor with men. Regularly, I fall in love. I dream a lot of being in love. (35 years)

Experiences with sexual abuse were reported by three participants. These experiences included sexual assault, rape, sexual abuse and online sexual betrayal. In all of these cases, the perpetrator was a man. These experiences had a great impact on the lives of the women. Two participants had multiple experiences with sexual abuse. These traumatic episodes and experiences still influence their daily lives. They struggle with fear, negative feelings and depression.

…assaults at work…touching my breasts…. The ex of my mother didn’t keep his hands to himself…. Therefore, I can’t express my feelings with a man. I became sick. (40 years)

For many years, I had online contact - on Facebook - with a beautiful woman. But it turned out to be fake. It was a man. I felt very bad about it. I couldn’t sleep for a long time. What a misery! I was heartbroken. I had a very bad time. Then I told my mom about it. We didn’t report this to the police. There was too little evidence. (25 years)

I have had many nasty experiences with sex…Yes, I was raped. I have flashbacks…. I want sex, but I don’t dare… the fear is stronger…. (38 years)

### Mental Health

Six out of ten participants had experienced mental health problems and have struggled with extreme loneliness, depression, alcohol addiction, anxiety, and being bullied. These six women talked openly about the difficulties in their lives, how they had tried to deal with them, and the mental health problems that can arise.

For a long period, I had a bad time. I wasn’t well, mentally. I have autism, got depressed, I stayed in bed for a year. Then I received psychological treatment and moved to another group home. There, the owner of the particular group home stole my money, approached me aggressively…. So, I moved on again… I went to live with my mother. That was not easy at all. (25 years)

I lived on my own and had my own apartment….and I was very lonely…. I recently suffered from a serious depression…and can’t stop thinking… I have appointments with a psychiatrist. (25 years)

Positive thinking is the best…Otherwise I start brooding….I can worry a lot….then I go to my doctor… he just talks to me and we laugh a lot…. (25 years)

I have a lot of grief…In my life many unpleasant things have happened… I drank a lot…. Now not anymore…. (38 years)

The participants received support to cope with these problems from family members and professionals such as paid care staff, psychiatrists, or general practitioners.

### Social Contact

Family members are important when it comes to social contact. Six participants talked about contacts with siblings: four participants described good contact with their family members; two participants talked about negative experiences and no longer have contact with their siblings.

My sister was the first who knew (lesbian feelings). I asked her to tell my parents. And she did. They responded well. (35 years)

My sister…no contact…She annoys me. My brother-in-law too. (34 years)

Alongside siblings, others family members were referred to: parents, children, aunts, a niece and grandparents. The reaction of one grandmother was:

If you are gay, you are no longer my little baby. (38 years)

The parents of eight participants reacted in a positive way:

…she (mother) always said it was good when I fall in love with a girl (29 years)

Two participants were unsure about the reaction of their parents:

I never mentioned it to my father. He always said: ‘Gay people are dirty’. (25 years)

Nine participants reported having a good relationship with friends. They talked about a circle of friends and social activities. How large and stable the circle of friends was, was not always clear.

Due to my autism, it is hard to make contact… I can be very lonely… Now I have two very good friends… I have to force myself to get (socially) involved. (25 years)

…I occasionally get comments…Sometimes I do get sad…Someone with whom I get along well…I do not feel happy. (25 years)

Seven participants lived in a care complex at the time of data collection: one lived in a group home and six lived in a private apartment in a care complex. Many have therefore had to deal with roommates or neighbours who live in the same care complex. Four participants talked about the reactions of their housemates, and the social contact they have with them. This contact was usually restricted to moments when they had a cup of coffee together.

I have mentioned it… but I don’t know if they understand it. They don’t ask. I have the feeling that they do not understand me…. I keep it to myself. (35 years)

…It is none of their business. (36 years)

And, as previously mentioned, nine participants mentioned how their friends responded to their coming out.

### Discrimination

Of the ten participants, seven women talked about their—mostly negative—experiences with being openly lesbian. In the first instance, they reported that they experienced no discrimination: “Nowadays, everything is very different…we can do whatever we like…” (40 years). Thereafter, they gave examples that in fact demonstrate the opposite:

Quite often, foreign people don’t accept us…We don’t feel at ease…I don’t find it important if people know who we are… (38 years)

In society it’s normal… a man with a woman, or a woman with a man. People act weird. They avoid me… I feel it. (35 years)

They say: within ten years you will have a man… They don’t take me seriously…Sometimes, it makes me sad. (25 years)

Two women talked about how they hide their sexuality.

You cannot see that I am bisexual. (36 years)

We walk side by side…never hand in hand. (38 years)

Despite the difficulties these women encountered, six out of ten women were open about their sexuality at work and in day programs.

She was bullied at work… they shouted: ‘lesbian, lesbian, lesbian!’ And they threw wads of paper at her. If it was up to me… I would beat them. (38 years)

They can say whatever they want…. (34 years)

## Discussion

This study used in-depth interviews to investigate the experiences of ten women with an ID who reported that they are lesbian or bisexual. We were interested in the lived experiences of lesbian and bisexual women with a mild ID in the Netherlands. To our knowledge, no such a study of this kind has been carried out before. Sexual experiences—and specific problems relating to sexual orientation as well as support needs—were explored in a semi-structured interview setting. Six general themes emerged in our data: support, coming out, sexual experiences, mental health, social contact, and discrimination.

Women with an ID need to interact with professionals providing support in relation to various life domains. When it comes to sexuality, it has been shown that relatives and support workers have different standards for themselves than for people with ID [33–35]. As a result, it is difficult for participants to talk to others about sensitive subjects such as their sexuality. They must often seek information without any support or input from others. Some of the participants we interviewed received support and guidance from the LGBT community. Coming out (revealing your sexual orientation) is not easy when you have an ID. All participants in our study were not used to standing up for themselves, often felt insecure, and had low verbal skills. Generally, with regard to having sex with another woman, most women had to figure out everything by themselves. This corresponds with the findings of Dinwoodie et al. [35]. This lack of support, feelings of insecurity, the lack of empowerment, and the limited verbal skills, hinders them to be self-conscious and find a partner. Six out of ten participants reported that they had experienced difficulties in finding a partner. Although the positive reactions of friends encouraged them in their ‘coming out’ process, they lacked a role model. This had affected their sexual experiences. Eight women reported that they had to find out almost everything by themselves. Three participants reported having been sexually abused by a male perpetrator. Social contact tended to be limited to relatives and friends. Other social networks such as those provided by work, leisure clubs and neighbourhoods were hardly mentioned.

The experiences of our participants are consistent with those previously reported in the literature (mainly in reference to gay men with an ID) and include negative perceptions, negative experiences, failures in sex education, exclusion, discrimination, and harassment [7, 10, 12, 14, 22, 24, 35]. Additionally, participants in our study reported various mental health problems, including feelings of depression, and anxiety. This corresponds with the outcome of research by Clarke et al. [16], who reported that LGBT individuals without an ID are also at increased risk for mental health problems.

### Supporting Women with ID

Providing support to lesbian or bisexual women with an ID is an essential part of securing their sexual rights. To do this, it is necessary to provide support in the following three domains: (1) sex education and training, (2) social contact, and (3) assertiveness. Naturally, in order to provide proper and adequate support, it is important to listen carefully to the wishes and needs of each individual, and to pay attention to individual circumstances and constraints.

#### Sex Education and Training

When it comes to providing sex education for people with intellectual disabilities, it is important to take into account particular learning disabilities, and any limitations in adaptive skills, which may make it harder to understand and remember information. In other words, sex education should be tailored to the particular needs of individuals. To proactively pay attention to the positive aspects of sex [36], rather than providing sex education only when there are problems, or in relation to potential problems, is also of value. There is hardly any information available for women with an ID who are lesbian or bisexual that provides answers to their specific questions or focuses on the pleasurable aspects of sex with another woman [11, 14, 22, 28].

#### Social Contact

Social contact is important in terms of experimenting with relationships, gaining experiences, developing a positive self-image, and avoiding loneliness. In this study, we found that participants’ social contact was mainly limited to relatives and friends. To meet a (sexual) partner, it is necessary for LGBT women meet other LGBT women. Therefore, it would be helpful to provide support that focuses on developing social skills, enabling access to transport, increasing opportunities to meet others, and developing the financial ability to undertake social activities [37]. This would contribute directly to quality of life. To provide support to individuals navigating their way in the LGBT community, is also important. Facilitating peer support can mean that support is provided in an accessible and approachable manner, and it can reduce feelings of isolation [35]. Commitment is required from the LGBT community—by connecting the specific needs of these women—, from caregivers and family members. Releasing everyone’s own normative framework is essential to match optimally to the wishes and needs of these women.

#### Assertiveness

Loshek and Terrell [38] point to the positive link between sexual assertiveness and positive physical and mental health outcomes for women. They distinguish three dimensions of sexual assertiveness in their sexual assertiveness questionnaire: the ability to initiate and communicate about desired sex, the ability to refuse unwanted sex, and the ability to communicate about sexual history and risk. For women with ID who are lesbian or bisexual, it is important to expand sexual assertiveness and to become resilient towards homophobic peers, caregivers, teachers and parents. Unfortunately, people with ID are often stigmatized [39–41], and those who are lesbian, gay or bisexual have to face a double stigma [11, 19, 22, 35]. Therefore, it is important that precisely these women (women with ID who are lesbian or bisexual) are supported so that they can become more sexually assertive and learn to cope with this double stigma. Only in this way, can these women practice their sexual rights as provided defined in the UN convention [3].

### Social Awareness

Women with ID are part of our society and organizational systems. Richards et al. [42] state that organizational systems, families, support staff, agencies, schools, and government have a social assignment to establish a climate of openness and attention to the sexuality of people with ID. Richards et al. [42] advocate a holistic approach ‘…in which all systems and supports work cohesively, simultaneously, seamlessly, and in harmony to ensure that persons with developmental disability achieve equality in sexual rights…..working together to support the individual with ID and takes a positive approach to ensuring that their sexual rights and needs are met’ [42, p. 210]. An inviting attitude of all is necessary for this vulnerable group: women with ID who are lesbian or bi-sexual. Only then, they can develop themselves and claim a full-fledged place in our society.

#### Limitations

There are several reasons to be cautious when interpreting the results of this study. First, the number of participants is limited. To recruit enough participants for this study, was a difficult assignment. Support providers judged that some women with ID were too vulnerable to be interviewed. Some women decided not to participate. They did not want to talk to a researcher about such a sensitive subject. Or intermediaries did not know any women with ID who are lesbian or bi sexual. Second, participants were interviewed only once, about a sensitive topic and in a short amount of time. The results would have been richer if there had been more interviews with every participant. In this way, our participants would have become accustomed to the researcher, perhaps resulting in more intimate conversations and more reliable data. Because people with ID have a strong tendency to seek approval, and a tendency towards acquiescence in interviews, there is a strong likelihood that the data will be distorted [43, 44]. As to the methodology, the reliability of this study would have increased when the analyses were conducted by two authors. Lastly, in this study, we interviewed a specific cohort of women with ID. Namely, women with a mild ID who were willing and able to talk about a sensitive topic with an unknown researcher. This sample is not representative of all lesbian and bisexual women with ID in the Netherlands.

#### Recommendations

The population of people with ID is very heterogeneous; sexual diversity also exists within this group. In order to affirm, defend and respect the sexual rights of these individuals, we would like to make some recommendations.

First, sexuality is an important life domain. More effort should be made to increase awareness about sexual diversity in people with intellectual disabilities. Efforts to raise awareness should focus particularly on those who support individuals with an ID (such as staff, counsellors and teachers). The aim is for LGBT women with ID to become more self-aware and less vulnerable, and to able to use all possible resources and methods made available to them. Intellectual disability services and the LGBT community should implement policies to actively include and support LGBT people with ID. In addition, those developing and providing sex education should ensure that sexual education and training programs are developed in a systematic way and in line with the latest scientific research [45, 46]. Intellectual disability services and (special education) schools should monitor the quality of these sex education programs. And finally, additional research is needed to learn more about sexuality and people with ID, in particular LGBT women with ID. Research institutions and funding bodies must prioritise research that focuses on this minority group in order for their fundamental human and sexual rights to be met. Research can contribute to a better and more fulfilling life for lesbian and bisexual women with ID.

## Appendix

Interview schedule/topic list

### Coming Out

- What makes somebody lesbian?
- Are you lesbian/someone who loves women?
- How did you find out?
- Do you talk about it with others? With whom?

### Setting/Environment

- How does your family respond?
- How do the people at your work respond?
- How do facilitators respond?
- Have you experienced unpleasant incidents?
- Discrimination? Pointing? Abuse?

### Sexuality

- What was your first experience? How old were you then?
- Do you have a partner?
- Do you have sex with people without intellectual disabilities?
- Do you get gifts or money for sex?
- Have you ever had sex with a man?
- Where do you have sex? And when?
- What is masturbation? Do you ever masturbate?
- Where? Is it allowed?
- Can you have sex freely? Without interference from supervisors? In your own room? Can you lock the door?
- Do you have sex only when you want it? Or do you have sex against your will?
- What is an STD?
- What can you do about it?

### Lifestyle

- Do you know other lesbians?
- Do you have contact with other lesbians?
- Do you visit bars for lesbians? Which? Why?
- Can anyone see/notice that you’re lesbian? How?
- Is there anything you would like to change in your life? What?
- Where are you proud of?

### Evaluation Interview

- How was the interview?
- Which questions did you like?
- Which questions were difficult?
- Were there questions I shouldn’t have asked?
- Are there any questions I forgot?

## Footnotes

1 COC is a LGBT organisation in the Netherlands; COC stands for ‘Cultuur en Ontspannings Centrum’ (Culture and Recreation Center); which is a name from the time that homosexuality was not as accepted as it is today.
